# The Impact of *SNCA* Variations and Its Product Alpha-Synuclein on Non-Motor Features of Parkinson’s Disease

**DOI:** 10.3390/life11080804

**Published:** 2021-08-09

**Authors:** Luca Magistrelli, Elena Contaldi, Cristoforo Comi

**Affiliations:** 1 PhD Program in Clinical and Experimental Medicine and Medical Humanities, University of Insubria, 21100 Varese, Italy; magis.luca@gmail.com; 2 Movement Disorders Centre, Neurology Unit, Department of Translational Medicine, University of Piemonte Orientale, 28100 Novara, Italy; elena.contaldi@aslvc.piemonte.it; 3 PhD Program in Medical Sciences and Biotechnology, University of Piemonte Orientale, 28100 Novara, Italy

**Keywords:** Parkinson’s disease, alpha-synuclein, non-motor symptoms

## Abstract

Parkinson’s disease (PD) is a common and progressive neurodegenerative disease, caused by the loss of dopaminergic neurons in the substantia nigra pars compacta in the midbrain, which is clinically characterized by a constellation of motor and non-motor manifestations. The latter include hyposmia, constipation, depression, pain and, in later stages, cognitive decline and dysautonomia. The main pathological features of PD are neuronal loss and consequent accumulation of Lewy bodies (LB) in the surviving neurons. Alpha-synuclein (α-syn) is the main component of LB, and α-syn aggregation and accumulation perpetuate neuronal degeneration. Mutations in the α-syn gene (*SNCA*) were the first genetic cause of PD to be identified. Generally, patients carrying *SNCA* mutations present early-onset parkinsonism with severe and early non-motor symptoms, including cognitive decline. Several *SNCA* polymorphisms were also identified, and some of them showed association with non-motor manifestations. The functional role of these polymorphisms is only partially understood. In this review we explore the contribution of *SNCA* and its product, α-syn, in predisposing to the non-motor manifestations of PD.

## 1. Introduction

Parkinson’s disease (PD) is the second most common neurodegenerative disease, and its prevalence tends to increase in an age-dependent manner [1]. With a general increase in life expectancy, PD is one of the world’s fastest growing neurological disorders, currently affecting about 1% of the population above 65 years [2]. PD causes severe disability in patients and a consequent psychological burden in caregivers [3]. Moreover, the concurrence of high prevalence and disability impacts on the cost of care, contributing to a significant economic burden [4]. Most PD cases are sporadic, while only 5% of PD patients present a genetic form, and mutations in LRRK2 gene are the most frequently detected [5]. The disease is clinically defined by the presence of bradykinesia, rest tremor and muscular rigidity, which, at the beginning, are unilateral, but subsequently spread contralaterally during the disease course [6]. In addition, patients complain of several non-motor symptoms (NMS) that may even precede for years the onset of the motor phenotype [7]. These include, among others, hyposmia, constipation, depression, pain and, in later stages, cognitive decline and dysautonomia [4]. Recent studies have shown that NMS have an even greater impact on patients’ disability and caregivers’ distress compared to the motor counterpart [8]. For such reasons, the spectrum of NMS has recently gained immense attention, and the last generation of clinical trials have incorporated NMS as important endpoints [9]. Furthermore, NMS take part in the fluctuations affecting patients in the intermediate-advanced disease phases, when the pharmacological regimen does not cover the daily functional requests and complex therapy modifications or advanced strategies are required [10]. It is therefore important for clinicians to recognize and monitor these symptoms using the already validated clinical scales [11].

PD pathology is characterized by loss of dopaminergic neurons in the midbrain substantia nigra with consequent accumulation of Lewy bodies (LB) in the surviving neurons [12]. The main component of LB is represented by the protein alpha-synuclein (α-syn), whose aggregation and accumulation perpetuates neuronal degeneration [13]. A-syn is expressed not only in the central nervous system, where it represents about 1% of all cytosolic proteins, but also in peripheral regions, contributing to the great variety of non-motor symptoms commonly seen in PD patients [14].

There is great interest regarding the roles of α-syn both in physiological and pathological conditions, especially considering that current treatment of PD relies only on symptomatic therapies, essentially aimed at restoring dopamine transmission, while the search for disease modifying strategies so far has been elusive.

A-syn is composed of 140 amino acids and has three different regions: the amino-terminal portion, which is important for the α-sheet structure, the central hydrophobic structure (called NAC), which confers the potential ability to assume a β-sheet conformation and the carboxy terminal part [15].

The diverse functions of α-syn are not yet completely understood. A-syn is detectable in the pre-synaptic area, near the synaptic vesicles, and may be involved in synaptic plasticity. Moreover, α-syn can be released in the synaptic space in part through the exosomes. This extracellular component plays an important role in neuronal homeostasis and may be involved in cell death [16]. The toxic effect of α-syn is due to its fibrillation and consequent accumulation. This is a multistep and exponential process determined by the formation of intermediates, in which the protein assumes a beta sheet conformation, which is enhanced by several factors such as oxidative stress, lipids, membranes, certain pesticides and metals [17]. Furthermore, some point mutations of the SNCA gene confer a higher fibrillation tendency, accounting for a younger age at onset with a more severe clinical phenotype [18].

Recent evidence indicates that α-syn is recognized by immune cells, and such interaction may have important pathogenic implications. T lymphocytes specific for α-syn epitopes were detected, and their frequency was higher in PD patients compared to controls [10,19]. In addition, an association between PD risk alleles at the HLA locus and anti- α-syn T cells was reported [19]. Such findings suggest that α-syn may drive the immune system towards a pro-inflammatory response [20], which may, in turn, impact PD motor and-non motor features [21,22,23,24].

Mutations in the α-syn gene (SNCA) were the first genetic cause of PD to be identified [25]. Several point mutations, as well as gene multiplications (duplication and triplication), have been described. Generally, patients carrying SNCA mutations present early onset PD with severe and early non-motor symptoms, including cognitive decline [26]. Moreover, SNCA presents interactions with other known PD causative genes (such as LRRK2, DJ1, PINK1 and Parkin). In particular, α-syn and LRRK2 co-localize in the LB. This phenomenon is already detectable in the early disease phases in the midbrain region, and tends to extend to the cortical regions as disease progresses [27]. DJ1 interacts with soluble monomeric and oligomeric forms of α-syn. Overexpression of DJ1 reduces α-syn dimerization, whereas mutant DJ1 causes an impairment of this process [28]. PINK1 interacts with α-syn, inducing its degradation and preventing its association with the mitochondria, which leads to cell death. Mutations or deletions of *PINK1* contrast these actions, increasing α-syn toxicity [29]. Parkin activates phosphatase A2, which in turn de-phosphorylates α-syn, thus attenuating cell death and inflammation. Conversely, α-syn counteracts this molecular pathway, enhancing cell loss [30].

In this review, we discuss the contribution of SNCA and its product, α-syn, in the non-motor manifestations of PD.

## 2. Genetic Variation of SNCA and Non-Motor Features

SNCA has six exons and encodes for α-syn, a protein of 140 amino acids. Due to alternative splicing, different transcripts can be generated and one of these, which is composed of 112 amino acids, and therefore called SNCA112, is particularly found in Lewy bodies [31].

### 2.1. SNCA Mutations

Pathogenic point mutations, few of which have been described, account for slightly different clinical phenotypes. Particularly, PD patients carrying the A53T and the E46K mutations have an early-onset disease with severe parkinsonism and dementia, while those carrying the A30P mutation have a less severe phenotype [32]. Tambasco et al. compared clinical presentations of patients with gene multiplications: triplications implied a more severe burden of non-motor symptoms compared to duplications, including higher prevalence of depression, psychosis, gastrointestinal and urinary dysfunctions and postural hypotension. On the contrary, anxiety and sleep disturbances were equally represented in the two groups, as well as in point mutation carriers [33]. The higher frequency of dysautonomia among triplication carriers was also confirmed by Singleton and colleagues, who demonstrated the presence of postural hypotension and cardiac sympathetic denervation after a few years of disease. Furthermore, some of these patients presented urinary or fecal incontinence [34]. Gene duplication also causes severe and rapid cognitive decline. Kielb et al. recently described a patient with an SNCA duplication who presented an initial impairment in executive and frontal/subcortical functions, which deteriorated more rapidly than the motor symptoms, ultimately leading to a Lewy bodies dementia phenotype with cognitive fluctuations, visual hallucinations and REM sleep behavior disorder [35].

SNCA mutations were also studied for their ability to modulate the immune response, especially considering that detrimental immune activation may influence PD features and progression [36]. Interestingly, SNCA mutations preferentially drive pro-inflammatory pathways. Carriers of the A53T mutation display higher production of the pro-inflammatory cytokine IL1α [37]. A subsequent study confirmed the role of the A53T mutation, but also showed that A30P and E46K mutations had an even more robust capacity in inducing a pro-inflammatory response in microglia [38].

Single nucleotide polymorphisms (SNPs) consist of a nucleotide change in the gene sequence that can occur both in intronic or coding regions [39]. Small polymorphic repeats represent the repetition of subsequent motifs, which can be classified as short tandem repeats (STR) if the repeating unit is composed of up to six base pairs, or variable number tandem repeats (VNTR) if the unit has more than six base pairs [40]. All these variations may influence protein expression and therefore be implicated in disease pathogenesis [39]. Several SNCA SNPs and polymorphic repeats have been examined, both in terms of disease predisposition and progression, but data are still controversial [41].

### 2.2. Rep1

Rep1 is a polymorphic dinucleotide repeat sequence located about 10 kb upstream of the SNCA transcription start site. This repeat is triallelic (259, 261 and 263 base pairs in length) and the 263 bp allele has a higher frequency in patients than in controls [42]. The length of this polymorphism directly correlates with protein expression. In particular, the 261 bp risk allele enhances gene transcription increasing protein levels, and its presence is therefore associated with a higher risk of developing PD. By contrast, the 259 bp risk allele lowers protein expression, exerting a protective role [43]. Moreover, Rep1 may act indirectly through the interposition of other transcription factors. Particularly, Poly (ADP-ribose) polymerase-1 (PARP-1) is a DNA binding protein able to modulate gene transcription and mediate several cellular pathways involving DNA duplication and repair, cancer and apoptosis. PARP-1 can bind Rep1 and, through a molecular mechanism not yet fully understood, actively modulate SNCA expression [44]. To further support the notion that Rep 1 length influences disease susceptibility, Shu et al. reported that Rep 1 alleles 265, 269 and 271 conferred an increased risk whereas allele 267 conferred a reduced risk of PD development [45].

As regards the influence of Rep 1 on non-motor features, evidence is rapidly growing. In a large cohort of 1134 Chinese PD patients, the prevalence of depression, assessed with the Hamilton Rating Scale for Depression (HADS), was significantly lower in carriers homozygous for the (CA)12 allele of the copy number variation of the SNCA Rep1 allele [46]. Results were later confirmed in a group of 171 patients from Singapore, where Rep1 longer allele (263 bp) carriers had a higher burden of non-motor symptoms investigated with the total score on the Non-Motor Symptoms Scale (NMSS) [47]. Furthermore, the authors showed that 263 bp allele carriers had a higher frequency of depression and fatigue, according to the Fatigue Severity Score [47].

Longer Rep 1 alleles were also associated with cognitive decline. In a cohort of 426 Italian PD patients, 263 bp genotype carriers displayed a significantly increased 5-year cumulative risk of dementia and visual hallucinations [48]. Findings were later confirmed in a Singapore cohort of 172 PD patients, where significantly lower scores on the Mini Mental State Examination (MMSE) in long versus short allele carriers were detected [49]. In both cohorts, Rep1 263 carriers also displayed a worse motor phenotype, but findings were robust only in the Singapore cohort, where both UPDRS part III and Hoehn and Yahr scores differed significantly in longer versus shorter allele carriers. Most studies found that patients carrying longer Rep1 alleles had an earlier age at onset, suggesting that the effect of the repeat on cognitive performance is independent from age [45]. On the other hand, there are also a few studies that failed to detect an association between Rep1 alleles and cognitive deterioration. In a large cohort of more than 900 patients, Markopoulou and colleagues found that patients carrying longer alleles did not display worse cognitive outcomes than patients with shorter Rep1 alleles [50]. These results are in line with a previous study performed by the same group in a Greek family showing that the 259-bp Rep 1 allele had a severe phenotype with a poor clinical outcome in terms of both motor and non-motor symptoms [51]. Nonetheless, significant methodological differences between the reported studies should be considered. First, clinical data included in the Italian study were collected through direct patient examinations, whereas data in the report by Markopoulou et al. were obtained through telephonic interviews [50]. Second, the way findings were displayed in the two studies makes the results difficult to compare. The report by Markopoulou et al. did not directly assess the impact of the 263 bp allele, but aggregated patients with 261–261 and 259–263 genotypes into the same group [50].

### 2.3. SNCA SNPs

Other SNPs of SNCA were evaluated with respect to cognitive function in PD. In a population of about 100 PD patients and 100 healthy controls from Brazil who were tested with MMSE and Frontal Assessment Battery (FAB) and genotyped for SNCA rs356219 and rs2736990, it was found that variations of both SNPs were associated with the risk of dementia. The most striking effect was seen in carriers of G allele of rs356219 in both homozygosis (GG) and heterozygosis (GA), who showed an odds ratio of 4.47 and 5.74 of developing dementia, whereas a weaker though significant impact on cognitive decline was displayed by CT and CC carriers at rs2736990, who had an odds ratio of 3.87 and 3.84, respectively [52]. Dementia development was also investigated in a cohort of European or North American ancestry composed of 1492 PD patients, 922 Lewy body dementia patients and 971 healthy controls. Assessing cognitive abilities with the Movement Disorders Society criteria and the MOCA, authors found that the C haplotype of rs62306323 and T haplotype of rs7689942 predicted dementia in PD patients [53]. More recently, carriers of G allele at rs356219 were also found to display cognitive impairment combined with faster motor progression compared to non-carriers [54]. Finally, a robust association between rs894280 and cognitive decline, particularly affecting attention and visuospatial functions, was detected in a group of 101 PD patients from Canada. Intriguingly, the authors used a computer-based approach, consisting of an informatics algorithm combining imaging, genetic and clinical features to identify the determinants of global cognition [55].

SNCA expression is also influenced by post-transcriptional modifications, such as methylation, consisting of the attachment of cytosine in a CpG dinucleotide site, mostly located in the promoter region of genes [56]. Generally, hypermethylated sites determine a lower gene expression by reducing the DNA polymerase binding, therefore interfering with the transcriptional process. SNCA presents these sites in the promoter region and in intron 1, and it has been suggested that modifications in the methylation process may influence SNCA levels and consequently have an important impact on disease susceptibility [56]. In this regard, Iakovenko et al. analyzed the methylation status of SNCA in a group of 460 PD patients and controls. Authors found a significant correlation between intron 1 site methylation and Rep1 allele length, with longer alleles correlating with hypomethylations. In contrast, no correlations were detected with promoter sites and in controls [57].

As regards other non-motor manifestations, depression, hyposmia and REM sleep behavior disorder (RBD) are particularly interesting for their frequent presence in the prodromal phase of the disease. Prevalence of depression was found inversely correlated with the TT genotype of SNCA rs2583988 in Brazilian PD patients [52]. Hyposmia and RBD were studied in a murine model of PD, characterized by the concomitant presence of SNCA A53T mutation, rs11931074, rs3857059 and Rep1 (259 to 261 alleles). Authors showed that in experimental mice, loss of dopaminergic neurons, hyposmia and RBD preceded the onset of the classical motor features of PD [58].

## 3. Alpha-Synuclein Production, Deposition and Non-Motor Features

The mechanisms through which common SNCA polymorphisms participate in PD pathogenesis still need to be completely elucidated [15]. Common genetic variations can modulate the alternative splicing process that leads to the generation of different SNCA transcripts and interfere with protein expression in central and peripheral tissues [59].

SNCA transcripts were investigated in a group of nine PD patients and six controls, and transcripts 112 and 98 were found increased in the cerebellum of patients compared to controls [60]. SNCA112 lacks exon 5, and that structural difference may enhance protein aggregation due to a significant shortening of the unstructured C-terminus [61]. G alleles of rs356219, rs365165 and rs2736990 have an important impact on SNCA112 production, possibly playing a detrimental role in PD [61]. On the other hand, studies showed that Rep1 genotype 259/259 causes lower expression of α-syn in both central nervous system, namely midbrain and temporal lobe [62], and peripheral blood mononuclear cells (PBMCs) [63].

3′ UTR SNPs influence central protein expression. For example, rs356219 may alter the generation of alternative splicing isoforms [64]. Moreover, AA and CT genotypes of rs356219 are associated with higher levels of SNCA mRNA in human substantia nigra and temporal lobe, respectively [62,63]. Linnertz and colleagues detected higher α-syn levels in midbrain in carriers of A haplotype of rs365165 [62]. A study of the gastric and colonic mucosa performed in 38 PD patients and 46 controls showed that patients who carry the G allele of rs11931074, but not Rep1, display α-syn deposition in the enteric nervous system [65]. The results are summarized in Table 1.

A-syn is also expressed in erythroid cells, both in bone marrow and in peripheral circulating cells [66]. Locascio and colleagues analyzed the expression of SNCA transcripts in circulating blood in a cohort of about 200 PD patients and controls. Expression of SNCA transcripts was significantly lower in patients than in controls, showing a 17% decrease. In particular, de novo patients displayed the most abundant transcript reduction. SNCA undergoes alternative splicing, generating different isoforms that can be detected in peripheral blood [67]. Accordingly, Marsal-Garcìa et al. studied the expression of five different SNCA transcripts in patients with PD and Lewy body dementia (LBD). Peripheral blood mRNA expression of transcripts 1 and 2 was reduced in patients with disease onset before 70 years while transcript 3 was increased in early PD. Furthermore, their concentration increased with disease duration [68]. Considering that LBD is a synucleinopathy characterized by early cognitive deterioration, a significant difference in SNCA transcripts between PD and LBD patients may represent a candidate marker of faster cognitive decline [68].

## 4. Peripheral Accumulation of α-Synuclein and Non-Motor Symptoms

*SNCA* undergoes several post-translational modifications, such as phosphorylation, ubiquitination and nitration, which may impact tissue protein expression and ability in aggregating, contributing to cell death [69]. According to the Braak hypothesis, pathological α-syn gradually reaches the CNS, particularly the basal ganglia, from the olfactory bulb and the vagus dorsal nucleus, and follows a subsequent diffuse cortical spreading. At the same time, α-syn is expressed in several peripheral tissues [70]. Such peripheral α-syn deposition was recently studied in a cohort of PD patients compared to matched healthy controls. Protein concentrations were analyzed in samples from skin, colon (particularly the sigma portion) and submandibular gland along with serum and CSF. About 25% of skin biopsies of PD patients and no controls were positive for phosphorylated α-syn, thus suggesting that the skin is an accessible and specific tissue for α-syn detection and therefore a support for PD diagnosis [71].

### 4.1. Gastrointestinal Symptoms

Constipation represents one of the most frequent, and often disabling, non-motor symptoms that can precede for years the onset of the clinical symptoms [72]. Accordingly, α-syn can be detected in the colon submucosal tissue and in the submucosal plexus in both ascending and descending colon [71].

Furthermore, patients may complain of sialorrhea and dry mouth, which often may coexist. In these disabling conditions, conventional medical treatment often is not sufficient and botulinum toxin injections are necessary with good results [73]. Pathophysiologically, α-syn can accumulate both in the submandibular gland and minor glands [71]. Interestingly, α-syn accumulation in the submandibular gland tends to increase with disease progression [74].

Dysphagia represents a life-threatening symptom because of the risk of aspiration, and therefore pneumonia, which mainly involves PD patients in the advanced phases of the disease [75]. Accordingly, α-syn has been detected in the cervical portion of vagus nerve and pharyngeal plexus [76].

### 4.2. Hyposmia

Hyposmia is a frequent non-motor symptom that precedes by several years the onset of the motor phenotype [77]. It is related to the accumulation of α-syn in the olfactory system; it has been detected not only in the olfactory bulb and tract but also in the cortex and specifically in the anterior olfactory nucleus and olfactory cortex [78]. Interestingly α-syn was present not only in neurons but also in non-neuronal cells (in decrementing order, microglia, pericytes and astrocytes) but not in the oligodendrocytes [79].

### 4.3. Cardiovascular Symptoms

About 30–40% of PD patients present orthostatic hypotension, defined as a fall in systolic blood pressure of at least 20 mm Hg or diastolic blood pressure of at least 10 mm Hg within 3 min when changing position from supine to standing [80]. The exact pathophysiology of cardiovascular involvement in synucleinopathies is complex and not fully understood. Cardiovascular symptoms are mainly expressed as myocardial noradrenergic deficiency due to denervation [81] and impairment of catecholamine turnover, which may cause the accumulation of toxic metabolites, leading to cell death [82]. Aggregates of α-syn have been detected in the sympathetic cardiac fibers [83], as also demonstrated by Isonaka and colleagues who studied a colocalization index of α-syn and tyrosine hydroxylase (TH) as an indicator of innervation. The authors found that all LB patients had an index > 1.5, indicating an accumulation of α-syn along with a neuronal denervation [84]. Cardiovascular symptoms may also be related to baroreflex failure, which may determine an exaggerated response to vasoactive therapies [85]. Last but not le least, orthostatic hypotension may also be related to anti-parkinsonian therapy [86].

### 4.4. Visual Impairment

Most PD patients present visual deficits (such as decreased visual acuity, abnormal spatial contrast sensitivity and color vision defects) along with retinal abnormalities [87]. α-syn aggregates have been identified in retina, particularly in the inner nuclear and ganglion layers where also dopaminergic receptors (particularly D1 receptors) are expressed. These aggregates can be found in both the axon and soma [88]. Furthermore, the increased latency detectable through the visual evoked potentials revealed that visual impairment is not only limited to the retina but involves the visual pathway [88].

### 4.5. Peripheral Neuropathy

PD patients may present a peripheral neuropathy, with both an acute/subacute and chronic onset [89]. It is still controversial whether PD may itself represent a risk factor or neuropathies may be correlated with the pharmacological treatment; accordingly, patients treated with continuous LD intestinal infusion have a higher incidence probably because of vitamin malabsorption (vitamin B12 and folate) with consequent accumulation of metabolites (such as methylmalonic acid) that may damage peripheral nerves [90]. In this context, α-syn aggregates and a higher α-syn ratio, which indicates the protein deposition in relation to nerve density, have been identified within pilomotor and sudomotor fibers [91] and in the unmyelinated fibers of the dermis [92]. These findings seem quite specific for PD because they have been detected in a small percentage of patients with atypical parkinsonism and in no controls.

All these data suggest that peripheral α-syn aggregates may represent a key factor leading to NMS development in PD. Nevertheless, the precise mechanisms through which such aggregates cause tissue damage and consequent NMS have not yet been demonstrated [93].

## 5. α-Syn in Other Neurodegenerative Diseases

Even though the main neurodegenerative diseases, including Alzheimer’s disease (AD), Huntington disease (HD) and amyotrophic lateral sclerosis (ALS) present very different clinical phenotypes, they may share some similarities in terms of the pathophysiological pathways involved. Working on such a hypothesis, the role of α-syn has been investigated in all of these conditions. The presence of α-syn and therefore Lewy bodies was detected in more than 50% of brains of AD patients [94]. Generally, α-syn has a higher tendency to co-localize with tau rather than with β amyloid. Particularly, α-syn may increase tau phosphorylation, which may in turn contribute to amyloid aggregation and accumulation, perpetuating a vicious cycle. Furthermore, higher levels of α-syn in the cerebrospinal fluid (CSF) of patients with mild cognitive impairment correlated with higher probability of progression to AD [95]. HD is caused by a trinucleotide expansion in huntingtin (HTT) gene. A-syn can promote the accumulation of mutated htt, both when expressed in wild-type form or in the presence of pathogenic mutations such as A53T and A39P, by increasing the aggregation rate of a part of *HTT* exon 1, which contains the expanded region [96]. Furthermore, α-syn may also influence disease features; in a murine model, symptoms such as tremor and weight loss were strictly correlated with α-syn levels [97]. Finally, ALS consists of a progressive, often rapid, degeneration of first and second motor neurons. Though rare, some cases are genetic, and the first mutated gene detected was the superoxide dismutase 1 gene (SOD1) [98]. Both in vitro and in vivo experiments showed that SOD1 presented a higher oligomerization rate in the presence of α-syn. Moreover, α-syn can act both on wild-type and mutated SOD1 [99]. All these data corroborate the need to further investigate α-syn involvement, not only in PD, but also in other neurodegenerative diseases, and to explore whether this protein might be a target for disease-modifying therapy.

## 6. Conclusions

**Figure 1:**
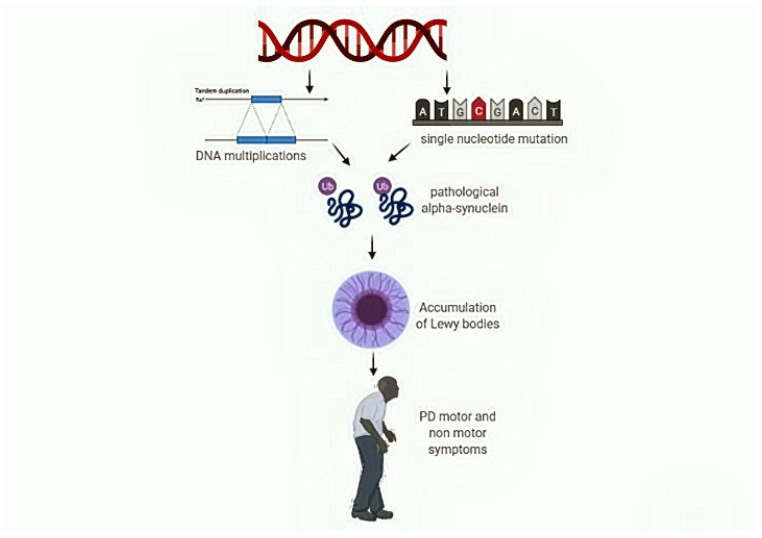
The impact of SNCA variation in the development of non-motor symptoms in Parkinson’s disease. Gene mutations and polymorphisms generate different SNCA transcripts. A-syn accumulates in the Lewy bodies or circulates in body fluids. Its interaction with immune cells drives a pro-inflammatory response ultimately favoring cell death. A-syn overproduction, spreading and deposition contribute to motor and non-motor manifestations.

Current evidence indicates that α-syn is of paramount importance in PD pathogenesis. Gene mutations are very rare but represent the first proof of the central role of this protein in PD. SNCA genetic polymorphisms certainly have a role in both modifying the risk of disease development and predisposing to peculiar phenotypic features, especially in the non-motor domain. Whatever lies beneath the variable degree of α-syn aggregation and accumulation in both CNS and peripheral tissues, it is clear that there is a solid correlation between such phenomena and clinical features of PD, especially regarding non-motor symptoms. Furthermore, α-syn spreading may drive the immune system toward a pro-inflammatory status, perpetuating the neurodegenerative process. (See Figure 1).

For such reasons, α-syn represents a promising therapeutic target for disease-modifying approaches in PD. Starting from the gene level, where SNCA multiplications increase α-syn production, promising results were obtained with RNA interference, providing a significant decrease in α-syn deposition in murine hippocampal neurons [100]. The major argument lies in the ideal target reduction, considering that a decrease of more than 90% of α-syn expression led to nigrostriatal degeneration [101]. A-syn decrease can also be achieved by acting on SNCA transcription. Recently, beta2 adrenoceptor agonists have been investigated as possible treatment strategies in PD [102] because they can modulate histone deacetylase action in gene promoter and enhancer regions, with consequent neuroprotective activity both in vitro and in murine PD models [103]. Particularly, clenbuterol was able to reduce α-syn mRNA and protein levels in mice in a dose-dependent manner. Moreover, clenbuterol administration in SK-N-MC human cells decreased α-syn levels previously boosted by propranolol administration. The neuroprotective action of clenbuterol may be ascribed to its ability in reducing acetylation of histone 3 lysine 27 (H3K27), a promoter of SNCA transcription [103]. Furthermore, several lines of research have studied the inhibition of α-syn aggregation by enhancing the heat shock protein functions [104] or by using oligomer modulators that can inhibit α-syn oligomer formation and accumulation, without reducing protein levels [105]. Levin et al. demonstrated that one of these modulators was able to slow the disease course in a PD murine model carrying the A30P mutation [106]. Last but not least, immunotherapy may be exploited to remove α-syn oligomers and deposits in PD, using an approach analogous to that already tested with beta-amyloid in Alzheimer’s disease [107]. Both active and passive immunization protocols provided encouraging though very preliminary results in PD. In particular, the administration of monoclonal antibodies provided a decrease in plasma α-syn concentrations and counteracted the cortical spreading of the protein [108]. Because phase I trials revealed that these drugs are safe and well tolerated, phase II and III are still ongoing. Furthermore, other molecules are under investigation; four studies are now targeting α-syn reduction both with immunotherapy and with small molecules that inhibit protein aggregation. Memantine, an NMDA receptor antagonist commonly used for dyskinesia treatment, is now being evaluated for its ability in counteracting α-syn cell-to-cell transmission [9].

## Figures and Tables

**Table 1:** Effects of SNCA SNPs on non-motor symptoms and gene expression.

SNP	Population	Results	Citation
Effects on non-motor symptoms			
rs356219 rs2736990	100 PD patients and 100 controls	Carriers of G allele of rs356219 had higher probability of developing dementia	[52]
rs2583988	100 PD patients and 100 controls	TT genotype inversely correlated with depression	[52]
rs62306323 rs7689942	1492 PD patients 922 Lewy body dementia and 971 controls	G allele of rs62306323 and T allele of rs7689942 were predictors of dementia development	[53]
rs356219	50 PD patients	G allele carriers presented a cognitive impairment along with a faster motor decline	[54]
rs894280	101 PD patients	CC genotype of this SNP was associated with cognitive decline	[55]
Effects on gene expression			
rs356219 rs365165 rs2736990	117 healthy subjects (brain samples)	Important impact on SCNA112 production	[61]
rs365165	144 healthy subjects (brain samples)	Carriers of A allele had higher alpha-synuclein levels in midbrain	[62]
rs356219	144 healthy subjects (brain samples)	Carriers of AA genotype had higher mRNA SNCA levels in substantia nigra	[62]
rs356219	17 PD patients and 24 controls	Carriers of CT genotype had higher mRNA SNCA levels in temporal lobe	[63]
rs11931074	38 PD patients and 46 controls	Carriers G allele of rs11931074 had enteric deposition of alpha-synuclein	[65]

## Data Availability

Not applicable.
